# Emerging roles of centrosome cohesion

**DOI:** 10.1098/rsob.220229

**Published:** 2022-10-26

**Authors:** Hairuo Dang, Elmar Schiebel

**Affiliations:** ^1^ Zentrum für Molekulare Biologie der Universität Heidelberg, Deutsches Krebsforschungszentrum-ZMBH Allianz, and; ^2^ Heidelberg Biosciences International Graduate School (HBIGS), Universität Heidelberg, Heidelberg 69120, Germany

**Keywords:** centrosomes, centrosome cohesion, centrosome linker

## Abstract

The centrosome, consisting of centrioles and the associated pericentriolar material, is the main microtubule-organizing centre (MTOC) in animal cells. During most of interphase, the two centrosomes of a cell are joined together by centrosome cohesion into one MTOC. The most dominant element of centrosome cohesion is the centrosome linker, an interdigitating, fibrous network formed by the protein C-Nap1 anchoring a number of coiled-coil proteins including rootletin to the proximal end of centrioles. Alternatively, centrosomes can be kept together by the action of the minus end directed kinesin motor protein KIFC3 that works on interdigitating microtubules organized by both centrosomes and probably by the actin network. Although cells connect the two interphase centrosomes by several mechanisms into one MTOC, the general importance of centrosome cohesion, particularly for an organism, is still largely unclear. In this article, we review the functions of the centrosome linker and discuss how centrosome cohesion defects can lead to diseases.

## Graphical abstract

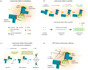

## 1. Introduction

The centrosome is a non-membrane bound organelle present in animal cells that functions as the main microtubule-organizing centre (MTOC) [1,2], meaning it has the ability to initiate microtubule (MT) polymerization and to anchor MTs [3,4]. It was originally discovered in the late nineteenth century by Theodor Boveri and Edouard Van Beneden. During their study of embryonic division in the nematode Ascaris, they identified the role of the centrosomes for mitotic spindle organization and cell division, as well as its self-duplicating ability [5].

The core of the centrosome consists of the mother centriole, composed of nine MT triplets. These triplet MTs are relatively stable compared to the cytoplasmic or spindle MTs, due to posttranslational modifications i.e. polyglutamylation and acetylation, as well as bound proteins that cross-link MT triplets [1]. Centrioles provide structural integrity to centrosomes and are surrounded by a proteinous material, named the pericentriolar material (PCM). PCM proteins such as pericentrin (PCNT), CEP192 and CDK5RAP5/CEP215 extend from centrioles into the cytoplasm and regulate MT assembly and centriole duplication [6]. Centrosomes facilitate MT assembly by the recruitment and activation of the γ-tubulin ring complex (γ-TuRC) that promotes *de novo* assembly of MTs from αβ-tubulin subunits [1,7]. Through MT organization, the centrosome regulates the shape, polarity and motility of cells and the formation of the mitotic spindle [2]. Centrioles duplicate once per cell cycle in a semiconservative manner commencing from G1 phase by a scaffold-based mechanism, starting with the recruitment of the polo-like kinase 4 (PLK4) by the proteins CEP192 and CEP152 to the outside wall of the two mother centrioles. PLK4 then recruits the centrosomal proteins CEP85 and STIL (SCL/TAL1 interrupting locus) [8,9] followed by the assembly of the Sas-6 (Spindle assembly defective-6) cartwheel [10] and the formation of daughter centrioles in S phase.

**Figure 1.**
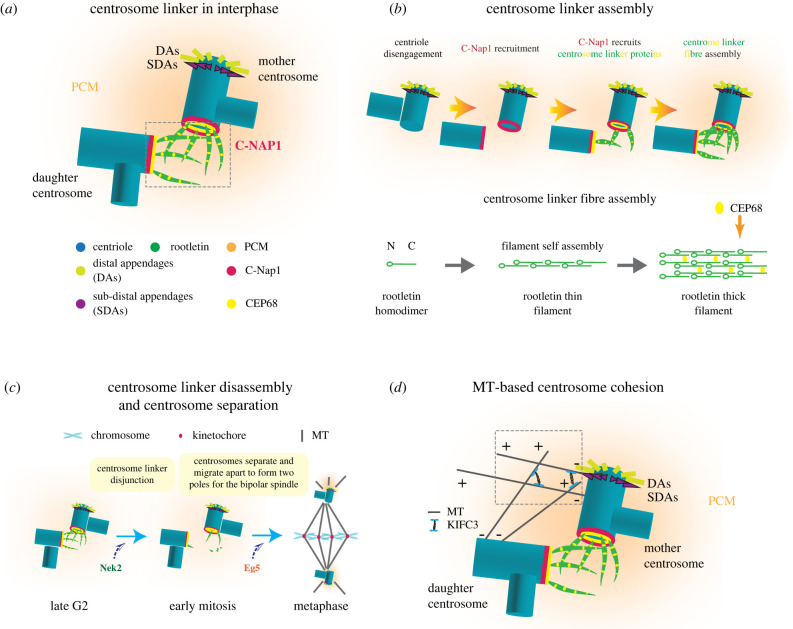
The centrosome linker and MT-based centrosome cohesion. (*a*) Molecular composition of the centrosome linker in interphase. C-Nap1 docks to the proximal end of the centrioles forming a ring-like structure that anchors centrosome linker proteins such as CEP68 and rootletin. Rootletin and CEP68 form the interwoven filaments of the centrosome linker. Abbreviations: DAs, distal appendages; SDAs, subdistal appendages [32]. (*b*) An overview of centrosome linker assembly. The centrosome linker protein C-Nap1 anchors rootletin filaments to the proximal end of centrioles. CEP68 interacts and stabilizes the rootletin fibre [32,146]. (*c*) An overview of centrosome linker disassembly and centrosome separation. Disassembly of centrosome linker allows centrosome separation. The separated centrosomes migrate to form the opposite poles of the bipolar spindle. MTs, microtubules. (*d*) MT-based centrosome cohesion in interphase. KIFC3 cross-links MTs organized by SDAs of the mother and PCM of the daughter centrosomes, thus creating pulling forces that keep the centrosomes together during interphase [20].

Centriole duplication is tightly regulated and its dysregulation can trigger defects in spindle formation and chromosome segregation leading to aneuploidy, the occurrence of aberrant chromosome numbers. Abnormal centrosome numbers are frequently associated with cell transformation and known as one important character of cancer cells [1,11]. Following centriole duplication in S phase, the daughter centriole, which is still attached to the mother, matures into a centrosome until the end of mitosis/G1 by recruiting PCM proteins and then becomes disjoined from the mother by separase cleavage of PCNT during mitotic exit [12,13]. Thus, G1 cells have, according to the assembly time, an older and younger (former daughter) mother centriole that both carry PCM and therefore function as centrosomes. Of the two centrioles in G1 cells, only the older mother centriole contains distal (DAs) and subdistal appendages (SDAs). These structures are subsequently acquired on the younger mother centriole shorty before or at the end of the next mitosis dependent on the DA and SDA proteins. Importantly, in interphase cells, the DAs of the mother centriole are responsible for centriole docking to the plasma membrane and thereby forming a primary cilium, a signalling and sensing organelle [4,14,15]. The SDAs of the mother centriole more stably bind MTs compared with the MTs organized by the PCM of centrosomes [16–18] (figure 1*a*).

## 2. Molecular mechanisms of centrosome cohesion

After centrosome duplication, the two centrosomes of an interphase cell are connected by at least two mechanisms, the centrosome linker [19] and the MT pathway [20], into one MTOC. These mechanisms work synergistically to keep the centrosomes together as one MTOC until the onset of mitosis when the two centrosomes become disjoined ensuring proper bipolar spindle formation.

### 2.1. The centrosome linker

The centrosome linker is mainly composed of the proteins C-Nap1 (centrosomal Nek2-associated protein 1, encoded by *CEP250*), rootletin (encoded by *CROCC*) and CEP68. Additional linker proteins, LRRC45 (leucine-rich repeat-containing 45), centlein (CNTLN), β-catenin and CCDC102B (coiled-coil domain containing 102B), have been described [21–25]. LRRC45 was reported in Hela cells as a centrosome linker component that associates with the proximal end of centrioles via C-Nap1 and whose depletion causes centrosome splitting [21]. However, interestingly, a recent investigation showed that in the non-cancerous cell line RPE1, the appendage proteins CEP83 and SCLT1 recruit LRRC45 to the mother centriole where it has a function in ciliogenesis and not in centrosome linker formation [26]. These phenotypic differences indicate that LRRC45 is differently involved in regulating centrosome cohesion and ciliogenesis in distinct cell types. Its precise functions and mechanisms await to be confirmed by further investigations. CNTLN was first described as a centrosome linker component that interacts with C-Nap1 and CEP68 and whose depletion causes centrosome separation [27]. Recently, the same group reported the role of CNTLN as an MT binding protein. Destabilization of MTs by CNTLN depletion could be the cause of the centrosome disjunction phenotype since this may inactivate the MT centrosome cohesion pathway [22]. It was reported that β-catenin forms a complex with rootletin and associates at the proximal end of centrioles dependent on C-Nap1 and rootletin [27]. However, this finding awaits confirmation by other groups. CCDC102B is recruited to centrioles by C-Nap1 and interacts with rootletin fibres [23]. Moreover, depletion of CCDC102B triggered a mild increase in centrosome splitting. Because of the unclear functions in centrosome cohesion, we do not further discuss LRRC45, CNTLN and β-catenin in this review.

The centrosomal protein C-Nap1 (encoded by *CEP250*), a large protein of 2442 amino acids, locates ring-like at the proximal end of the centrioles and serves as a central anchoring point for centrosome linker proteins, particularly rootletin and CEP68. In human cells, C-Nap1 is anchored to centrioles by binding to CEP135. However, *CEP135* KO cells still recruit C-Nap1 but with reduced efficiency, indicating alternative docking mechanisms [28]. Nevertheless, both knockdown [29] or KO [28] of *CEP135* in cultured mammalian cell lines displayed centrosome linker defects. Intriguingly, *CEP135* KO in chicken DT40 cells seemed not to result in defects in centrosome cohesion [30], implying differences in the anchoring of C-Nap1 to centrioles dependent on the organism. Rootletin is an elongated coiled-coil protein (length of approx. 110 nm) that self-assembles into thin filaments [31,32]. CEP68 is a globular protein that binds via its C-terminal spectrin repeat-containing region to rootletin [32]. Rootletin, together with CEP68, forms highly ordered, repetitive and polar filaments. Within these filaments a rootletin molecule is shifted relative to its neighbour by 75 nm. Owing to the presence of one binding site in rootletin, CEP68 binds with a periodicity of 75 nm to rootletin fibres. CEP68 is not essential for rootletin filament formation. However, CEP68 assists in branching off rootletin filaments from centrioles and it modulates the thickness of rootletin fibres. The rootletin/CEP68 fibres form a flexible interwoven network that keeps the two centrosomes of a cell together. A multitude of low affinity interactions between rootletin/CEP68 filaments probably are the basis for the linker-based centrosome cohesion [32] (figure 1*a,b*).

The kinase NEK2 (NIMA Related Kinase 2) dissolves the centrosome linker in G2/prophase through phosphorylation of the linker components C-Nap1, rootletin and CEP68 [33–36]. This allows the two centrosomes to move apart and to organize the two poles of the mitotic spindle [37,38] (figure 1*c*). The centrosome linker reassembles with mitotic exit when the central anchoring protein C-Nap1 is dephosphorylated and then becomes attached to the proximal end of the mother and daughter centrioles by binding to the centrosomal protein CEP135 [29].

What is the function of the centrosome linker at the cellular level? Centrosome linker defects caused by knockout (KO) of the central centrosome linker gene *CEP250* have only mild consequences for a cell. Cell cycle progression and chromosome segregation are normal in linker-deficient *CEP250* KO cells. However, *CEP250* KO cells showed defects in the spatial organization of the Golgi apparatus and cell migration [33,39]. It is long known that centrosome derived MTs position the Golgi apparatus of a cell [40,41] and therefore it is easy to envision that the two separated centrosomes in *CEP250* KO cells each position spatially distinct Golgi stacks. In addition, in human and rodent cell lines, centrosome orientation was shown to be important to maintain polarization in migrating cells [42,43]. Studies in migrating *Dictyostelium discoideum* showed that the centrosome is located behind the cell′s leading edge [44] and repositioning of the centrosome stabilizes the direction of movement, probably via the MT system [45]. Thus, lack of centrosome coordination in centrosome linker-deficient cells, probably affects directed cell movement. Finally, a recently study showed localization of a group of SDA components at the proximal end of the centrioles via centrosome linker protein C-Nap1. SDA and C-Nap1 loss has no effect on the efficiency of cilia assembly, but disrupts stable cilia-Golgi association and switches cilia formation from a submerged intercellular location to the cytoplasmic membrane to form surfaced cilia that are exposed to the environment of the cell. Surfaced cilia respond actively to mechanical stimuli and signalling components (e.g. Hedgehog signalling components) even in absence of agonists, which consequently leads to disturbance in the direction of cell movement [46].

### 2.2. The MT pathway and actin in centrosome cohesion

Besides the centrosome linker as the most prominent element controlling centrosome cohesion in interphase, alternative centrosome cohesion pathways have been identified. A recent study described the human minus-end-directed, tetrameric kinesin MT motor protein KIFC3 in promoting centrosome cohesion. KIFC3 cross-links MTs derived from SDA of the mother centrosome and PCM of the daughter centrosome and creates forces that pull both centrosomes of an interphase cell together [20] (figure 1*d*). This centrosome cohesion mechanism becomes crucial in late G2 when the centrosome linker is already resolved by NEK2 kinase and the KIFC3/MT pathway first counteracts the increasing activity of the plus end directed tetrameric KIF11 (also known as Eg5) that pushes the two spindle poles apart [20]. Inactivation of the KIFC3/MT pathway by NEK2 is one factor that eventually determines the timing of mitotic spindle formation [18].

Besides being the main MTOC in animal cells, the role of centrosome in organizing actin filaments was also demonstrated in several recent studies, which showed centrosome was able to nucleate actin via the nucleation-promoting factor WASH and the Arp2/3 complex [47–49]. Centrosomal actin filaments were shown to form a physical barrier that inhibits nascent MT elongation. Consequently, reduction of actin filaments at centrosomes resulted in higher MT growth during cell adhesion and spreading in interphase [48]. Reversely, accumulation of centrosomal actin during anaphase is correlated with reduction in MTs at centrosomes [49]. These observations suggest important functions of centrosomes in regulating the crosstalk between actin and MTs, although the role of which in the context of centrosome cohesion remains yet not well understood. Nevertheless, importantly, several studies suggest that the centrosome attaches to actin filaments and MT regulators via the protein GAS2L1 (Growth Arrest Specific 2 Like 1), an MT- and actin-binding protein. This generates forces between centrosomes, which promote centrosome separation [50–53]. In addition, perinuclear actin was described to have a role in centrosome cohesion by antagonizing Eg5 forces emanating from the centrosomes in late G2/early prophase [54]. The interaction between the LINC (Linker of Nucleoskeleton and Cytoskeleton) complex at the nuclear envelope and the perinuclear actin was shown to be critical for this regulation, although the molecular mechanisms are less clear [55]. Taken together, cells have multiple and partly redundant mechanisms that promote centrosome cohesion.

## 3. Centrosome cohesion in development

### 3.1. Centrosome cohesion in early brain development

Mutations in centrosomal genes are frequently identified in autosomal recessive primary microcephaly (MCPH), a severe neuronal disorder characterized by smaller brain size and mental retardation [56,57]. MCPH is associated with reduction of neuronal populations during brain development and several genes were mapped to MCPH loci as the most frequent mutations [58–60] (table 1). Although the reason why centrosomal defects particularly impact brain development remains unclear, several studies indicate that many MCPH-associated genes are profoundly involved in centriole biogenesis e.g. *CEP135*, *CEP152* and *SAS-4* (group 1) or in generic functions of the PCM, e.g. *WDR62*, *ASPM1*, pericentrin and *CDK5RAP2/CEP215* (group 2) [61–64]. The first group (refereeing to loss of the genes *CEP135*, *CEP152* and *SAS-4*) results in centriole number alteration [29,65–67] and the second group (refereeing to loss of the genes *WDR62*, *ASPM1*, pericentrin and *CDK5RAP2/CEP215*) leads to MT nucleation defects and spindle abnormalities [68–71]. Mutations in these MCPH genes activate the mitotic surveillance pathway, namely the spindle assembly checkpoint [72,73] and p53 mediated apoptosis [74–77], which eventually impede the neural progenitor cell proliferation as the major cause of MCPH. Interestingly, studies have shown that inactivation of p53 restored brain size in mouse MCPH models with centrosome defects by promoting stem cell survival [74,78–80], nevertheless it did not restore asymmetric centrosome inheritance as typically seen in the wild-type (WT) stem cells [74].

**Table 1.** MCPH genes identified in human microcephaly. The table summarizes MCPH genes identified in human microcephaly [66–69,71,148,149].

MCPH genes	localization	other names	centrosomal function	reference
MCPH1	8p23	microcephalin	mitotic progression	Jackson *et al*. [148]
MCPH2	19q13.12	WDR62	PCM	Roberts *et al*. [68]
MCPH3	9q33.3	CDK5RAP2/CEP215	PCM, spindle assembly	Moynihan *et al*. [71]
MCPH4	15q21.1	CEP152	centriole duplication	Jamieson *et al*. [66]
MCPH5	1q31	ASPM	PCM, spindle assembly	Jamieson *et al*. [69]
MCPH6	13q12.2	CPAP, CENPJ, Sas-4	centriole duplication	Leal *et al*. [67]
MCPH7	1p32	STIL, SIL, Sas-5	centriole duplication	Cristofoli *et al*. [149]

**Figure 2.**
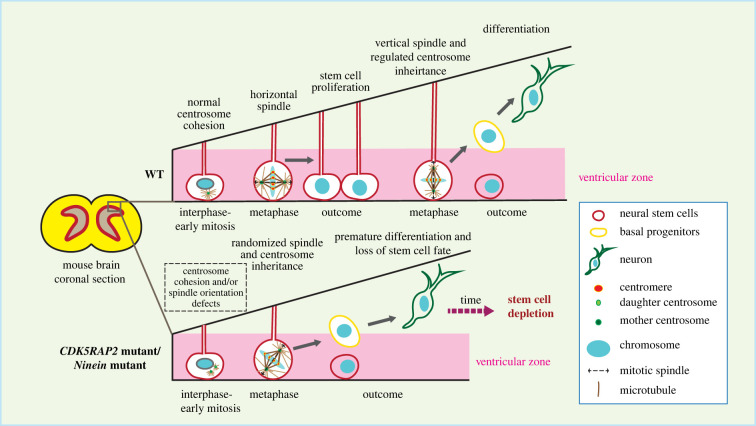
Genes involved in centrosome cohesion affect brain development. *CDK5RAP2/CEP215* or *NIN* mutations possibly lead to aberrant centrosome cohesion, resulting in dysregulated spindle orientation and asymmetric centrosome inheritance, triggering premature differentiation of the neural stem cells, which eventually results in loss of stem cells and reduced brain size [86–90].

In cells, *CEP135* and *CDK5RAP2/CEP215* depletion/mutation affect centrosome cohesion [15,28,29,81,82]. As discussed above, CEP135 functions as an anchoring point for C-Nap1 at centrioles and *CEP135* KO cells displayed centrosome linker defects [28,29]. *CDK5RAP2/CEP215* was originally described as a putative centrosome linker component. Later it became clear that CDK5RAP2/CEP215 is a regulator of the γ-TuRC [81,83–85] and its lack of function affects centrosome MT nucleation and therefore the MT-based centrosome cohesion pathway [15,62,86–88]. Another example is ninein (*NIN* gene)*,* a protein located at the SDA and the proximal end of the centrosome with functions in stable attachment of MTs to SDAs and therefore also MT-dependent centrosome cohesion [4,46]. The function of ninein in brain development was shown in several mouse studies, where they described the regulatory role of ninein in asymmetric cell division and self-renewing activities of the radial glia progenitors. *Nin* KO mice display an MCPH like phenotype with smaller brain and reduced stem cell pool [89–91] (figure 2). Since CDK5RAP2/CEP215 and ninein have dual roles in MT organization and centrosome cohesion, it is not clear which functional loss contributes to defects in brain development.

The observation that mutations in *CEP135*, *CDK5RAP2/CEP215* and *NIN* can lead to microcephaly raises the interesting question of whether *CEP250* defects also affect brain development? A study identified a homozygous nonsense mutation in the centrosome linker gene *CEP250* that impair centrosome cohesion and causes Seckel-like syndrome in cattle, a disease which is characterized by MCPH in addition to low body weight, hindlimb hypoplasia and skeleton dysplasia [92]. In addition, two genome-wide studies identified *CEP250* mutations in a small subset of east Asian populations where affected individuals showed mild reduction in height, however, without affecting brain development [93,94]. These data raise the possibility that *CEP250* and therefore the centrosome linker has a crucial role in development.

### 3.2. The essential role of centrosome linker in spermatogenesis

The Seckel-like syndrome phenotype of cattle with a homozygous truncation mutation in *CEP250* made it important to study the consequence of *CEP250* knockout in a mammalian model organism such as mouse. Two recent studies reported phenotypes of *Cep250* KO mice [95,96]. Dang *et al*. [95] used *Cep250* KO mice from gene deletion by Cre/LoxP system, while Floriot *et al*. [96] generated *Cep250* KO by the transcription activator-like effector nuclease TALEN. In both cases, the targeted mutations resulted in changes in first few exons of the *CEP250* gene, resulting in premature stop codons and short non-functional N-terminal C-Nap1 fragments, which do not localize to the centrosome [95,96]. Importantly, both manuscripts report that *Cep250* KO mice have defects in centrosome cohesion in the germline and male gametogenesis [95,96]. However, the MT centrosome cohesion pathway is still functional in *Cep250* KO MEFs as indicated by an increase in centrosome disjunction by the addition of the MT depolymerizing drug nocodazole. Thus, *Cep250* KO mice are only partially centrosome cohesion deficient [95].

Dang *et al*. [95] found that *Cep250* KO mice did not display significant defects in brain and body size, body weight and skeletal development, as compared to the *CEP250* mutant cattle. Histological analysis of the major tissues of *Cep250* KO mice also did not detect obvious defects although this analysis may not have tracked down minor defects. However, loss of *CEP250* resulted in a defect in germ stem cell (GSC) maintenance in the testis as early as P2, leading to depletion of germ cells and male infertility [95]. Dang *et al*. [95], showed an essential role of centrosome linker in spermatogenesis by controlling spindle orientation and asymmetric centrosome inheritance via facilitating the establishment of E-cadherin-based cortical polarity during male GSC division. In brief, in WT GSCs the centrosome linker keeps the two centrosomes in close proximity until G2/early mitosis. Such connection is required for the timely establishment of a polarity mark of the cell adhesion molecule E-cadherin on the cell cortex close to the basement membrane in mitosis, as seen by failure to establish such mark in *Cep250* KO GSCs. Although the exact mechanism by which the two adjacent centrosomes trigger the cortical enrichment of E-cadherin remains unclear, it is tempting to speculate that signalling molecules, particularly mitotic kinases at centrosomes, may signal to the cell cortex. Several kinases have been reported to play an important role in regulating cell polarity and asymmetric division (e.g. NEK kinases, CDK1, Plk1 and Aurora A) [25,97–99]. Therefore, it is very likely that that centrosome cohesion amplifies the transmitted signal from the centrosomes to the cell cortex by spatially combining both signalling centres.

**Figure 3.**
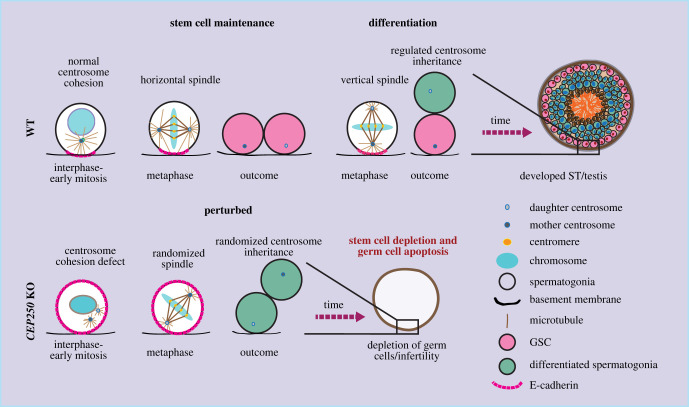
Centrosome cohesion in testis development. *Cep250* KO mice, which lack the centrosome linker, show defects in centrosome cohesion and premature centrosome separation in interphase-early mitosis. As a result, timely establishment of E-cadherin polarity pattern in early mitosis was disturbed, leading to dysregulation of spindle orientation and asymmetric centrosome inheritance. Centrosome cohesion defects consequently cause loss of the stem cell population and germ cell apoptosis [95].

The C-Nap1-dependent polarization of the cell cortex has two important outcomes. First, during early GSC development, the basal polarized E-cadherin directs the position of the two mitotic centrosomes to keep them horizontal relative to the basement membrane, thereby facilitating the establishment of a mitotic spindle that is oriented parallel to the basement membrane. This is crucial for the establishment of a vertical cell division plane and self-proliferation of the GSCs, thus the maintenance of the stem cell pool. By contrast, the defect in centrosome cohesion and in establishment of an E-cadherin polarity mark in *Cep250* KO GSCs leads to randomization of mitotic centrosome positioning and spindle orientation, which consequently triggers premature differentiation of the GSCs and failure to maintain the stem cell pool in testis. The prematurely differentiated cells move into the interior of the seminiferous tubules starting as early as P2, where they are eventually eliminated by apoptosis, leading to further reduction of the germ cell number. Additionally, the basal polarized E-cadherin also supports stem cell maintenance in later developmental stages by controlling the correct inheritance of the older (mother) centrosome to the cell remaining at the basement membrane after division, which maintains stem cell character. It does so by keeping the mother centrosome at a relatively close proximity to the basement membrane during mitosis. Such regulation is lost in the *Cep250* KO mice, resulting in failure in stem cell fate maintenance [95] (figure 3).

Interestingly, the role of asymmetric centrosome inheritance was described before in *Drosophila* male germline stem cells (GSCs) and mouse neural stem cells. The *Drosophila* GSC orientates its mother centrosome towards the hub cells (the stem cell niche in *Drosophila* testis) by polarization of E-cadherin and its interaction with adenomatous polyposis coli (APC) tumour suppressor homologue, which is important to maintain stem cell fate [100,101]. This is somewhat similar to the supportive function of basement-located Sertoli cells to spermatogenic cells in mouse testis [102–104], consistent with the importance of spatial temporal regulation of mouse GSC as discussed above. Comparably, in mouse brain stem cells the primary cilium anchors the mother centrosome of a neural stem cell to the apical membrane of the ventricle. Such mother centrosome anchoring is important for maintaining the behaviour and properties of the stem cell [89,105,106]. Here, the emerging picture is that organisms and tissues use different mechanisms and molecules to retain the mother centrosome close to the stem cell niche that maintains stem cell character. The situation in mouse testis reflects more *Drosophila* GSCs than mouse brain stem cells although the molecular players differ between *Drosophila* and mouse.

**Figure 4.**
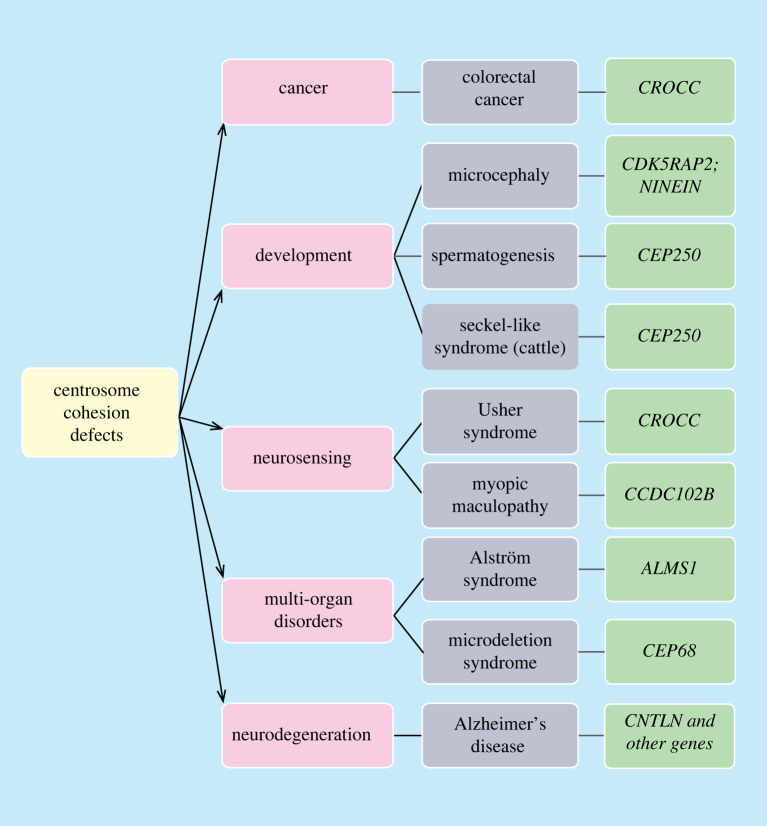
Diseases and disorders associated with centrosome cohesion defects. The diagram summarizes diseases (pink panels) and disorders (grey panels) associated with alterations of genes encoding known centrosome cohesion proteins (green panels) [86–90,92,95,112,117,119,120,123–126,131,133,147].

Floriot *et al*. [96] identified an additional function of *CEP250* in male meiosis. *Cep250* KO spermatocytes show abnormal meiotic progression as they are unable to progress through meiosis I [95,96]. These cells displayed aberrant γH2AX pattern (a marker for double-strand breaks during meiosis [107]) and arrested in pachytene stage. This results in accumulation of dysregulated spermatocytes in meiosis I, likely due to synapsis defects and the unrepaired DNA double-strand breaks [96]. These meiotic defects further escalate germ cell apoptosis. How the centrosome linker exactly affects meiosis I progression is presently not understood and requires further investigation.

One conceivable explanation of the phenotypic difference between cattle and mice can be the only partial loss of function of C-Nap1 in cattle (a truncation mutation in the *CEP250* gene) in comparison to its complete loss of function in mouse (homozygous gene deletion). In addition, distinct functions of the centrosomes in development among different species can also lead to incomplete recapitulation of human disorders in model organisms, which was demonstrated in numerous studies [108–110]. Importantly, loss of *CEP250,* hence the centrosome linker establishment, can be possibly compensated by alternative centrosome cohesion pathways that vary in their strength and activities in different tissues and organisms. Particularly, MT/KIFC3 dependent centrosome cohesion was previously shown to restrict the centrosome linker-based cohesion defect in cultured cells [20]. Such pathways may compensate the loss of the centrosome linker in other tissues than testis. Therefore, it will be interesting to study the phenotypes of *CEP250* and *KIFC3* double KO in mice particularly since loss of *KIFC3* does not have an obvious impact on mouse development [111].

## 4. Other functions of centrosome linker proteins

### 4.1. Centrosome linker proteins and neurosensory disorders

It is puzzling that spermatogenesis is seemingly normal in *Crocc* KO mice [112]. Divergences in the remaining linker function between *Crocc* KO and *Cep250* KO mice may account for the differences in spermatogenesis. For example, *Crocc* KO mice used in this study may not be full null since the inserted Neo^R^ marker did not disrupt the open reading frame of the *CROCC* gene [112]. In addition, the rootletin/CEP68 linker may function redundantly with alternative linker filaments that are also anchored to centrioles by C-Nap1. For instance, CCDC102B could be involved in the formation of such a centrosome linker structure [23].

However, *Crocc* KO mice showed reduction in vision as they age due to degenerated photoreceptors in photoreceptor cells [112]. The photoreceptor, which is a specialized cilium, is anchored to the nucleus by rootletin filaments that have a similar repeat organization than the rootletin filaments in the centrosome linker [113,114]. Although ciliary rootlets have no determinant roles in ciliary development and basal functions of the cilia, they are required for long-term maintenance of the cilia in photosensory cells [112] such that in ageing mice loss of *CROCC* leads to reduction of cilia length and stability, which ultimately result in decline in vision. This notion is consistent with the study on the nuclear envelope protein Nesprin1 in photoreceptor cells. Nesprin1α that associates with the nuclear envelope through a C-terminal KASH (Klarsicht, ANC-1, Syne Homology) domain binds to rootletin filaments of photoreceptor ciliary rootlets via a spectrin repeat (similar to CEP68 [32]) anchoring the cilium to the nuclear envelope [115]. Deletion of Nesprin1 resulted in similar phenotype as seen in *Crocc* KO, but at a much earlier timing of onset [116]. Intriguingly, *CEP250* mutations have been previously identified in Usher syndrome, a rare autosomal recessive disease which affects both hearing and vision [117]. The vision problem of Usher patients raises the possibility that C-Nap1 is also involved in photoreceptor ciliary rootlet organization. Such a defect will only become apparent in ageing mice and therefore would not have been detected in the two studies on *Cep250* KO mice that focused on developmental defects in young mice [95,96,112].

### 4.2. Centrosome cohesion associated with other genetic disorders

Genes that affect centrosome linker function have been identified in human heterogeneous genetic disorders meaning production of a single or collected phenotypes through different genetic mechanisms. For instance, mutations of *ALMS1*, which encodes a protein located at the proximal end of the centriole that recruits C-Nap1 probably together with CEP135, were seen in families with the rare genetic disorder Alström syndrome (figure 4). The affected individuals display various symptoms in multiple organs and body systems [118–120]. A recent study also identified *ALMS1* mutation in infants with dilated cardiomyopathy [121]. Yet it remains unclear whether *ALMS1* mutations alone can lead to the formation of these diseases. Nevertheless, *ALMS1* depletion in RPE1 cells (retinal pigment epithelial cells) showed shorter primary cilium length and downregulation of TGF-β signalling [122], which may explain the diseases due to defects in primary cilia function including signal transducing activities.

Indeed, many human disorders are triggered by mutations in multiple genes. Consistent with these complex scenarios of human genetic disorders, mutations in some centrosome linker genes have been identified in diseases associated with other genes. For example, mutations in *CEP68, CCDC102B* and *CNTLN* along with additional gene alterations, were identified in microdeletion syndrome, myopic maculopathy and Alzheimer's disease, respectively [123–125]. Likewise, *CROCC* mutation was also found in neuroblastoma patients [126]. These findings indicate the complexity of human genetic diseases, in which multiple regulatory processes are involved. Hence, centrosome cohesion may participate in many complex physiological processes. Further investigations are required including a more in-depth analysis of defects in tissues of *Cep250* KO mice, as well as studies of KO mice of other centrosome cohesion components. These studies are needed to understand how disturbance of centrosome cohesion can lead to perplexing changes in human health and development.

## 5. Centrosome cohesion in cancer

Structural and numerical centrosome aberrations are one important character of cancer and probably contribute to cancer development [127–130]. By contrast, the role of centrosome cohesion defects in cancer is largely unclear. However recent studies identified mutations in *CROCC* in several unusual and very aggressive colorectal cancer subtypes, which are referred to as the rhabdoid phenotype (figure 4) [131]. Although it is unclear how *CROCC* mutations contribute to the formation of these aggressive cancer subtypes, they were reported to cause chromosomal instability and chromosome segregation errors, which may trigger more severe cancer progression [131–133]. Indeed, the timing of centrosome cohesion/separation is important for spindle formation and correct chromosome attachment to spindle MTs [134,135]. It is possible that the mutant rootletin either has a dominant phenotype (e.g. in delaying resolution of centrosome cohesion at mitotic onset or *CROCC* mutations may work synergistically together with other mutations in colorectal cancer). The possibilities are not mutually exclusive.

In many transformed cells, centrosome number is elevated due to cytokinesis or centrosome duplication defects. Amplified centrosomes are clustered in mitosis after resolution of the centrosome linker in late G2 by the action of the MT motor proteins HSET (kinesin-14), Eg5 (KIF11) and dynein in order to prevent formation of a multi-polar spindle and chromosome miss-segregation [136–138]. This mitotic centrosome clustering ensures cancer cell survival and limits genome instability [139]. Several studies described inhibitors of HSET, Eg5 or dynein have a notable effect in reducing mitotic centrosome clustering in cancer cells with supernumerary centrosomes [140–144], hence sensitizing them for apoptotic death. Interestingly, amplified centrosomes are also clustered in interphase [119,145]. However, the relative contribution of the centrosome linker and the MT pathway to interphase clustering and whether this has an impact on supernumerary centrosome organization in mitosis is presently unclear.

## 6. Conclusion

Defects in centrosome cohesion and separation have been seen to affect numerous cellular processes, including timing of mitotic onset, spindle formation, cell polarity, motility, cellular transport and ciliation. Therefore, it is surprising to see that loss of the essential centrosome linker protein C-Nap1 in mouse has no impact on body and brain weight, or on the development of most organs, with the exception of testis, where sperm production was completely abolished [95,96]. Indeed, centrosome cohesion is a complex phenomenon, in which the centrosome linker and alternative centrosome cohesion pathways act in a synergistic and redundant fashion to ensure the linkage and correct separation timing of centrosomes. Distinct centrosome cohesion components may have different strength and activities in various tissues and organisms and dependent on this, loss of one of them may have phenotypic consequences. Nevertheless, centrosome cohesion defects in human health are much less studied as compared to centrosome aberrations in respect to numerical and structural changes. Hence, it is important to develop better tools that identify centrosome cohesion defects in the complex environment of a physiologically normal tissue or tumour and analyse phenotypes in models with loss of centrosome cohesion pathways, individually or in combination. Understanding the causes and consequences of defects in centrosome cohesion will shed light on our understanding of centrosome function as well as its potential in therapeutic treatment for human diseases.

## Data Availability

This article has no additional data.
